# Pelvic digit as a rare cause of chronic hip pain and functional impairment: a case report and review of the literature

**DOI:** 10.1186/1752-1947-3-139

**Published:** 2009-11-19

**Authors:** Marc Maegele

**Affiliations:** 1 Department of Trauma and Orthopedic Surgery, Intensive Care Unit, University of Witten/Herdecke, Cologne-Merheim Medical Center (CMMC), Ostmerheimerstr 200, D-51109 Cologne, Germany

## Abstract

**Introduction:**

Pelvic digit is a rare congenital anomaly where bone develops in the soft tissue adjacent to normal skeletal bone. The condition is benign and is usually discovered accidentally. On a plain radiography, pelvic digit typically appears as a rib- or phalanx-like bone structure with a clear cortex and medulla related to the pelvis, often with a pseudoarticulation at its base.

**Case presentation:**

We present the case of a 40-year-old Caucasian man who presented with chronic pain and tenderness over his right hip together with functional impairment in abduction and external rotation. Radiology identified a bony protuberance at the right anterior inferior iliac spine with fusion of the proximal bony nucleus to the adjacent bone. The pelvic digit was surgically removed and the patient was discharged free of symptoms and with complete range of motion in his right hip joint.

**Conclusion:**

It is important to recognize and distinguish a pelvic digit from post-traumatic ossification and avulsion to avoid unnecessary additional investigations.

## Introduction

Pelvic digit is a rare benign congenital anomaly where bone develops in soft tissues adjacent to normal skeletal bone [1]. The condition is usually asymptomatic and is often discovered accidentally [2]. However, pelvic digit may also cause pain and functional impairment and can cause some confusion in trauma cases, especially if the patient is symptomatic in the area [3]. Radiology characteristically shows a rib- or phalanx-like bone structure with a clear cortex and medulla related to the pelvis, often with a typical pseudoarticulation at its base [2]. We present the case of a 40-year-old Caucasian man who presented with chronic pain and tenderness over his right hip together with functional impairment. Radiology identified a bony protuberance at the right anterior inferior iliac spine with fusion of the proximal bony nucleus to the adjacent bone. The pelvic digit was surgically removed and the patient was discharged free of symptoms with complete range of motion in his right hip joint.

## Case presentation

**Figure 1:**
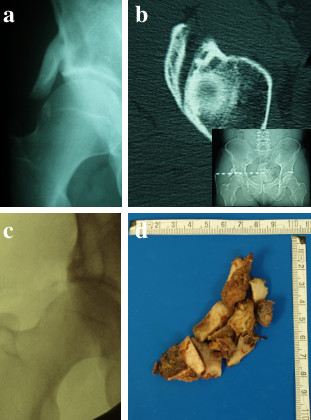
**Conventional radiography (a) and computed tomography including scout view (b) shows a bony protuberance at the right anterior inferior iliac spine**. Complete removal of the pelvic digit (c and d) restored the full range of motion within the patient's right hip joint together with complete relief of symptoms.

A 40-year-old white Caucasian man presented to our outpatient service complaining of right hip pain. He reported having experienced this discomfort since his youth with the condition now worsening, but he denied any history of trauma. Clinical examination revealed tenderness over his right hip together with functional deficits in abduction and external rotation in his right hip joint. Conventional radiography extended by computed tomography (CT) showed a bony protuberance at the right anterior inferior iliac spine with fusion of the proximal bony nucleus to the adjacent bone (Figure 1a and 1b). This finding together with the clinical picture was highly suggestive of a pelvic digit. Due to the patient's complaints including functional impairment in his right hip joint, surgical removal of the pelvic digit was performed (Figure 1c) and the patient was discharged 3 days later free of symptoms with a full range of motion in his right hip joint. Histopathological work-up was consistent with a rib bone (Figure 1d).

## Discussion

To date, few single cases and case series of pelvic digits have been reported. For example, Sullivan and Cornwell [4] described a 15-year-old girl with a well-defined 'rib' in the pelvis. The abnormal bone curved caudad towards the right side of the distal sacral vertebra but was not directly attached to the sacrum. Histological assessment after removal was consistent with a rib, as in our patient. The authors postulated that the abnormal bone originated embryogenically from the first coccygeal vertebra. A similar observation was made by Lame [5] who reported on the case of a 63-year-old man with a finger-shaped bony structure arising from the right iliac crest, terming this anomaly an 'iliac rib'. This author located the anomaly to the mesenchymal stage of bone growth before the sixth week of fetal development [6], with the posterior segment originating from a displaced rib center and the anterior counterpart from a displaced sternal center [5]. Morphologically, pelvic digits may present as rib-like [7] and phalanx-like structures with one or more (pseudo-) joints within [5-8]. Intermediate appearances with features of both rib and phalanx have also been reported [9]. The pelvic digit is most frequently attached to the ilium [2,3], but also to the sacrum [4], coccyx [10], abdominal wall [8], and, rarely, to the symphysis [6]. Although pelvic digits occur mostly unilaterally, bilateral occurrences have been reported [6].

There are some variations in the numbers of bony segments and (pseudo-) articulations of pelvic digits described in the literature. For example, Lame [5] and Granieri and Bacarini [7] described a total of six cases, all consisting of a bony structure of at least two bony elements and at least one (pseudo-) articulation. Nguyen *et al*. [8] reported a case series where one patient had one phalanx and one pseudoarticulation, and two other cases with three bony segments and two pseudoarticulations. A similar configuration was reported by Casey *et al*. [3].

Similarly, variable origins for the digits have been described. According to some authors, the anomaly can originate from a displaced costal process, a displaced sternal center, or the ossification center at the anterior superior iliac spine [4-6,9]. However, these suggestions do not reflect the sites of attachment in the pelvis, for example, at the coccyx, the pelvic walls, and the inferior abdominal wall. Therefore, it appears more likely that pelvic digits originate from an embryonic mesoderm with rib-forming capacity disposed to these regions [2]. At the end of the third week of embryogenesis, embryonic mesoderm cells with rib-forming capacity migrate from the primitive streak and pass around the cloacal membrane, finding their way from the region of the future coccyx via the region of the future pelvic walls, to the region of the lower abdominal wall [2]. In normal rib development, the posterior part of each rib originates as a 'costal process' of the mesenchyma, thus forming the vertebral centra [11]. It has been suggested that, in the pelvis, the 'costal processes' become incorporated into the lateral parts of the sacrum and coccyx [2]. The 'costal process' mesenchyma normally degenerates due to apoptosis. Absence of apoptosis may allow differentiation of 'costal process' mesenchyma into rib tissue. These bony structures may come into contact with the neighboring developing bone [2].

Differential diagnosis of a pelvic digit comprises myositis ossificans, avulsion, heterotopic bone formation, Fong's disease and osteochondroma. Fong's disease (onychoosteodysplasia) is a hereditary condition with dysplastic or absent nails and absent or hypoplastic kneecaps (nail-patella syndrome). Other characteristic features include iliac horns and abnormality of the elbows interfering with the full range of motion. In some cases, new bone formation after surgery or ossification of the sacrotuberous ligament can resemble a pelvic digit [2]. The pelvic digit is usually identified via radiography and differentiated from post-traumatic myositis ossificans and heterotopic bone formation by its corticated appearance in the absence of trauma [6,7,12]. Additionally, CT confirms the presence of cortical bone [3,8,13,14].

In the absence of clinical symptoms, surgical intervention is not required [12]. Our patient suffered from chronic pain along with functional impairment in his right hip joint. Diagnostic imaging including conventional radiography and CT revealed a right-sided pelvic digit that prompted surgical intervention. Complete removal restored the full range of motion within his right hip joint together with complete relief of symptoms.

## Conclusion

Pelvic digit is a benign congenital anomaly where bone develops in the soft tissue adjacent to normal skeletal bone. It is usually identified via radiography and differentiated from post-traumatic myositis ossificans and heterotopic bone by its corticated appearance in the absence of a traumatic event. Surgical removal is indicated in the case of symptoms such as functional impairment. It is important to recognize and distinguish a pelvic digit from post-traumatic ossification and avulsion to avoid unnecessary investigations.

## Abbreviations

CT: computed tomography.

## Consent

Written informed consent was obtained from the patient for publication of this case report and any accompanying images. A copy of the written consent is available for review by the Editor-in-Chief of this journal.

## Competing interests

The author declares that they have no competing interests.

## Authors' contributions

MM assembled all relevant data to this case report, performed the literature review and drafted the manuscript.
